# Insulin Resistance as a Link between Amyloid-Beta and Tau Pathologies in Alzheimer’s Disease

**DOI:** 10.3389/fnagi.2017.00118

**Published:** 2017-05-03

**Authors:** Roger J. Mullins, Thomas C. Diehl, Chee W. Chia, Dimitrios Kapogiannis

**Affiliations:** ^1^Laboratory of Neurosciences, Intramural Research Program, National Institute on Aging, National Institutes of Health (NIA/NIH)Baltimore, MD, USA; ^2^Translational Gerontology Branch, Intramural Research Program, National Institute on Aging, National Institutes of Health (NIA/NIH)Baltimore, MD, USA

**Keywords:** Alzheimer’s disease, insulin resistance, magnetic resonance spectroscopy, exosomes, IRS-1

## Abstract

Current hypotheses and theories regarding the pathogenesis of Alzheimer’s disease (AD) heavily implicate brain insulin resistance (IR) as a key factor. Despite the many well-validated metrics for systemic IR, the absence of biomarkers for brain-specific IR represents a translational gap that has hindered its study in living humans. In our lab, we have been working to develop biomarkers that reflect the common mechanisms of brain IR and AD that may be used to follow their engagement by experimental treatments. We present two promising biomarkers for brain IR in AD: insulin cascade mediators probed in extracellular vesicles (EVs) enriched for neuronal origin, and two-dimensional magnetic resonance spectroscopy (MRS) measures of brain glucose. As further evidence for a fundamental link between brain IR and AD, we provide a novel analysis demonstrating the close spatial correlation between brain expression of genes implicated in IR (using Allen Human Brain Atlas data) and tau and beta-amyloid pathologies. We proceed to propose the bold hypotheses that baseline differences in the metabolic reliance on glycolysis, and the expression of glucose transporters (GLUT) and insulin signaling genes determine the vulnerability of different brain regions to Tau and/or Amyloid beta (Aβ) pathology, and that IR is a critical link between these two pathologies that define AD. Lastly, we provide an overview of ongoing clinical trials that target IR as an angle to treat AD, and suggest how biomarkers may be used to evaluate treatment efficacy and target engagement.

## The Molecular Basis of Insulin Resistance

The binding of insulin to the insulin receptor leads to the recruitment and phosphorylation of the insulin receptor substrates 1 and 2 (IRS1 and 2; Draznin, 2006). These molecules represent the first node in the insulin signaling cascade, with further downstream nodes being phosphoinositide 3-kinase (PI3K) and protein kinase B (PKB/Akt), which in turn affect master regulatory switches of cell metabolism, cell survival, growth and differentiation, such as the mammalian target of rapamycin (mTOR), and glycogen synthase kinase 3 (GSK3; Pessin and Saltiel, 2000; Sarbassov et al., 2005; Tzatsos, 2009; Zhang and Liu, 2014).

Persistent activation of the insulin receptor results in excessive phosphorylation of Ser and Thr residues on IRSs (Czech et al., 1988; Singh, 1993; Tanti et al., 1994). This aberrant phosphorylation of IRS results in reduced insulin receptor binding sensitivity and translocation of the active portion of IRS from the membrane to the cytosol, and is one of the main molecular underpinnings of insulin resistance (IR; Aguirre et al., 2002; Boura-Halfon and Zick, 2009; Copps and White, 2012; Ryu et al., 2014). Moreover, these mechanisms have the potential for establishing pathogenic feed-forward loops that inhibit normal insulin signaling, as mTORc, ribosomal protein S6 kinase beta-1 (S6K1), and GSK3-β induce hyperphosphorylation at various Ser residues (S632, S302/S522 and S337, respectively; Eldar-Finkelman and Krebs, 1997; Copps and White, 2012).

A key physiological action of insulin is to increase glucose uptake into cells (Leney and Tavaré, 2009) by inducing translocation of various insulin-dependent glucose transporters (GLUTs) to the plasma membrane. GLUT-3 is the primary brain GLUT and is mainly expressed in axons and dendrites, but GLUT-1 and 4 are also expressed in the brain (Maher et al., 1991; Simpson et al., 2008). The uniquely low Michaelis-Menten constant of GLUT-3 allows for continuous transport of glucose into neurons even under low extracellular concentrations, thereby providing a consistent energy source (Duelli and Kuschinsky, 2001). Different isoforms of GLUT-1 mediate glucose uptake by astrocytes as well as the endothelial cells of the blood brain barrier (BBB). Interestingly, neurons in areas vulnerable to Alzheimer’s disease (AD; e.g., basal forebrain cholinergic neurons) show partial GLUT-4 dependence, which may help explain their vulnerability in low energy conditions and AD (Morgello et al., 1995; Apelt et al., 1999; Duelli and Kuschinsky, 2001). In systemic and organ-specific IR states, the ability of insulin to stimulate glucose uptake via GLUT transporters is impaired, requiring higher than normal concentrations of extracellular insulin to maintain normal glucose uptake to match cellular metabolic needs (Lebovitz, 2001).

## Brain Insulin and The BBB

While there is evidence that insulin is produced *de novo* in different brain regions, the general consensus remains that a majority of the insulin in the brain arrives from the periphery through the BBB (Pardridge et al., 1985; Kullmann et al., 2015), where it is concentrated to levels 50× higher than in circulating plasma independently of peripheral hormonal states (Havrankova et al., 1979; Banks et al., 2012; Blázquez et al., 2014). Peripherally produced insulin crosses the BBB via a saturable transport system, with partial saturation occurring at standard euglycemic levels (Woods and Porte, 1977; Banks, 2004). Peripheral insulin can enter the brain interstitial fluid (ISF) either directly through the BBB or via cerebrospinal fluid (CSF), but the relative contributions of each are not yet known (Genders et al., 2013). The levels of CSF glucose and insulin only partially reflect blood levels, suggesting their differential regulation in this compartment (Woods and Porte, 1977). In humans, the transfer of blood insulin into the CSF has been confirmed during intravenous injections of insulin (Wallum et al., 1987). Interestingly, in obesity the CSF/plasma insulin ratio is decreased, a finding that should be taken within broader context, as the CSF/plasma ratios for leptin and adiponectin are also decreased (Caro et al., 1996; Kos et al., 2007).

The BBB is a dynamic structure that homeostatically regulates the uptake and release rates for a variety of hormones, chemicals, and proteins (Daneman, 2012). Accordingly, fluctuations in plasma levels of both glucose and insulin affect their uptake by the BBB (Prasad et al., 2014). This uptake is carried out by the GLUT-1 and GLUT-3 transporters embedded within the BBB endothelium, providing the ability to respond to variable energy demands (Leybaert et al., 2007). This dynamic is demonstrated in a study that found glucose transport across the BBB increased with luminal expression of GLUT-1, whereas higher abluminal GLUT-1 expression was accompanied by decreased glucose transport (Cornford and Hyman, 2005). Insulin receptor expression is also reduced in the BBB when there is prolonged peripheral hyperinsulemia (Schwartz et al., 1990). The rate of insulin transport across the BBB is also slowed by obesity and aging. Obesity decreases the transport of insulin across the BBB, and this deficit can be reversed by starvation and caloric restriction (Urayama and Banks, 2008). Aging leads to an overall decrease in the number of insulin receptors at the BBB (Moreira et al., 2009). Insulin transport is diminished as a consequence, with CSF insulin levels being lower in both obese and older individuals (Heni et al., 2015). Insulin levels in the brain tissue of older individuals are also lower (Frölich et al., 1998). Additionally, decreased CSF levels of insulin correlate with poorer cognitive performance in patients with diabetes or AD (Moloney et al., 2010; Duarte et al., 2012).

Evidence also exists that insulin can be produced *de novo* in brain regions with many pyramidal cells, such as the hippocampus, prefrontal cortex, olfactory bulb and entorhinal cortex (Havrankova et al., 1978; Heidenreich and Gilmore, 1985; Marks et al., 1991; Devaskar et al., 1994; Mehran et al., 2012). While the significance of this evidence is still debated, recent studies show that functional insulin signaling components in forebrain regions may exert a neuroprotective role in areas responsible for various functions of memory (McNay and Recknagel, 2011; De Felice et al., 2014). Downstream elements in the signaling pathway known as the “PI3K route” have been shown to both promote neuronal cell survival and facilitate synaptic plasticity, providing a link between IR and AD (van der Heide et al., 2006).

## Brain Insulin Resistance

A variety of genetic, developmental, and metabolic factors underlie brain IR. Polymorphisms in the Fat Mass and Obesity-Associated Protein (FTO) gene, involving introns 1 and 2 that are highly expressed in the brain, exhibit strong effects on brain IR (Reitz et al., 2012). Carriers of the at-risk FTO-AA allele who are also carriers of an apolipoprotein-E (APOE) ɛ4 allele have a significantly increased risk for AD and dementia (Keller et al., 2011). Additionally, a single nucleotide polymorphism near the Melanocortin-4 Receptor (MC4R) gene, a gene expressed in brain regions that regulate systemic metabolism such as the hypothalamus (Shen et al., 2013), has been linked to increased brain IR (Tschritter et al., 2011). Moreover, maternal glucose and insulin sensitivity correlate with fetal brain responses to fluctuations in circulating glucose, suggesting a prenatal predisposition to brain IR (Linder et al., 2014). Increased circulating free fatty acids may also play a role in establishing brain IR. High fat diet (HFD) leads to rapid release of pro-inflammatory factors at the hypothalamus, and triggers the c-Jun N-terminal kinase (JNK) pathway to increase activation of the leptin and insulin signaling inhibitor nuclear factor kappa-light-chain-enhancer of activated B cells (NF-kB; Nakano, 2004; Sears and Perry, 2015).

Dysfunctional phosphorylation of IRS-1 has been extensively linked with brain IR, similar to other tissues. Total levels of insulin signaling proteins in the aforementioned “PI3K route” are not significantly different in the brains of AD patients vs. cognitively normal (CN) controls, suggesting that the phosphorylated active levels of these molecules are more relevant to IR and AD pathogenesis as opposed to total levels (Talbot et al., 2012). Studies in human hippocampal tissue have shown that phosphorylation mediated by factors such as mTOR and GSK-3β, coupled with feed-forward inhibition from the JNK pathway, leads to specific increased phosphorylation on multiple Ser residues of IRS-1 (specifically, S312, S616 and S636; Boura-Halfon and Zick, 2009; Fröjdö et al., 2009; Talbot et al., 2012). However, conflicting evidence exists showing that S307 phosphorylation in mice (human S312) may in fact increase insulin sensitivity and improve insulin signaling (Copps et al., 2010).

## Vascular Effects of Brain Insulin Resistance

Vascular function is tightly coupled to insulin signaling, and central to this relationship is endothelial dysfunction, which manifests through deficient vasodilation and improper vasoconstriction throughout the body in the setting of IR (Hsueh et al., 2004; Quiñones et al., 2004; Cersosimo and DeFronzo, 2006). The vasodilator effects of insulin are mediated by the PI3K signaling pathway, which leads to nitric oxide (NO) production in endothelial cells which elevates cyclic guanosine 3′,5′-monophosphate (cGMP) in vascular smooth muscle; insulin vasoconstrictor effects are mediated through endothelin-1 (Muniyappa and Quon, 2007; Muniyappa and Sowers, 2013). Insulin signaling causes a dose-dependent increase in NO production (Zeng and Quon, 1996), whereas impaired PI3K signaling decreases NO and cGMP, leading to decreased vasodilation (Francis et al., 2010). NO also inhibits platelet aggregation, monocyte adhesion, and thrombosis, all of which damage the vessel wall (Celermajer, 1997). Microvascular disruption leads to superoxide production, which, among other events, leads to a rise in advanced glycation end products. Pathological activation of the receptor for these advanced glycation end products (RAGE) increases oxidative stress, exacerbating vascular inflammation, thrombosis, and vascular damage (Kook et al., 2012). Impaired endothelial cell-mediated vasodilation may also be caused by excess free fatty acids (FFAs) traveling in the blood stream (Steinberg et al., 1997). FFA’s are often elevated in diabetic patients, and through the action of the inhibitor of nuclear factor kappa B kinase subunit beta (IKKB, which modulates NF-kB) inhibit the production of NO, decreasing vasodilation, deteriorating cardiovascular function, and exacerbating the insulin resistant state (Ginsberg, 2000; Kim et al., 2005).

Significant vascular pathology is frequently seen in older individuals with dementia. In fact, until the significance of neuritic plaques (NP) and neurofibrillary tangles (NFT) was unequivocally demonstrated, the prevailing view was that vascular pathology is primarily responsible for the cognitive deficits in AD (Kling et al., 2013). Vascular dementia is thought to be the second most common form of dementia after AD (Jellinger, 2007), whereas mixed pathology dementia is being increasingly reported in the literature, with more than half of all dementia cases being attributed to dual pathology (Langa et al., 2004; Schneider et al., 2007; Battistin and Cagnin, 2010). A variety of small and large vessel cerebrovascular disease pathologies have been described, including silent infarcts, leukoaraiosis (seen on magnetic resonance imaging (MRI) as white matter hyperintensities), cerebral amyloid angiopathy (CAA), microaneurysms, and small and large vessel ischemic/hemorrhagic stroke (Breteler, 2000; Gorelick et al., 2011; Attems and Jellinger, 2014; Corriveau et al., 2016). Recently, the term “vascular contribution of cognitive impairment and dementia” (VCID) has been coined to capture this heterogeneity.

There is emerging evidence showing that IR and diabetes have significant implications in VCID. It is well known that cerebral blood flow is decreased in diabetic patients (Jellinger, 2007). Cerebral small vessel disease (CSVD) is the cause of approximately 20% of strokes and the underlying etiology for many of the other pathologies previously mentioned (Lammie et al., 1997; Cai et al., 2015). Importantly, CSVD is aggravated by diabetes. Specifically, pathological hallmarks such as incident, small and large lacunes, and white matter hyperintensities seem to correlate with progression of IR (Dearborn et al., 2015). Diabetes also increases the risk for large vessel disease, and is present in approximately 30% of strokes (Karapanayiotides et al., 2004). A study showed that for each standard deviation increase in homeostatic model assessment for insulin resistance (HOMA-IR) and body-mass index (BMI), there was an increase in incident large lacunes. Moreover, higher IR score correlated with the increase in prevalence of both small and large lacunes (Dearborn et al., 2015). Increased HOMA-IR scores are associated with higher risk of ischemic stroke even among non-diabetics (Rundek et al., 2010). Interestingly, IR has been reported in almost half of non-diabetics who presented with a transient ischemic attack (Kernan et al., 2003).

## The Interplay of Insulin Resistance and Aβ Pathology

Several epidemiological studies have shown that the systemic IR state of type 2 diabetes is a major risk factor for age-related cognitive decline, dementia, AD, and progression from mild cognitive impairment (MCI) to AD (Ott et al., 1999; Arvanitakis et al., 2004; Li et al., 2016). Besides the aforementioned vascular contributions, several lines of evidence suggest that brain IR directly promotes the development of classic AD beta-amyloid (Aβ) and tau pathologies (Steen et al., 2005; de la Monte, 2012). Brain IR may also exacerbate pre-existing AD pathology by this same mechanism and is known to be associated with cognitive decline independently of AD pathology (Talbot et al., 2012; Umegaki, 2013).

Aβ refers to several peptides between 39–43 amino acids in length that are formed by the sequential β and γ secretase cleavage of the amyloid precursor protein (APP); a large transmembrane protein with an unknown physiologic role. Aberrant oligomerization of certain Aβ peptides (such as Aβ42) and formation of extracellular plaques with Aβ fibrils at their center in equilibrium with soluble oligomers are histopathological hallmarks of AD (Hardy and Selkoe, 2002; Blennow, 2004; Pearson and Peers, 2006; Greenwald and Riek, 2010). It has been shown that the distribution of regional glucose metabolism via glycolysis in normal young adults correlates spatially with Aβ deposition in individuals with AD, suggesting a pathogenic link between glycolysis in earlier life and eventual development of Aβ pathology (Phelps and Barrio, 2010; Vaishnavi et al., 2010; Vlassenko et al., 2010). Moreover, an important study found that regional lactate production is closely linked to interstitial Aβ levels, establishing an additional link between glycolytic energy metabolism and a key pathogenic protein in AD (Bero et al., 2011). Lactate is produced by astrocytes as a product of glycolysis and can be used as an alternate neuronal energy substrate in conditions that do not favor aerobic metabolism (Magistretti and Pellerin, 1999). More recently, elevated lactate in transgenic AD mice compared to wild type mice was seen *in vivo* and in association with memory deficits (Harris et al., 2016). A putative interplay between increased reliance on glycolysis, increased production of lactate and ensuing increased extracellular Aβ has the potential of establishing a feed-forward loop that perpetuates and aggravates Aβ pathology in AD.

It has been shown that insulin promotes brain Aβ clearance, preventing its extracellular accumulation and plaque formation (Watson et al., 2003). Conversely, IR promotes the formation of Aβ fibrils by inducing GM1 ganglioside clustering in presynaptic membranes (Yamamoto et al., 2012). Aβ oligomers increase activation of the JNK pathway, leading to increased IRS-1 pS616 (as well as Tau pS422; Yoon et al., 2012). Collectively, these data suggest a feed-forward loop where Aβ oligomers aggravate brain IR, which in turn decreases Aβ clearance and increases the propensity for Aβ oligomerization. Moreover, a recent study showed that Aβ oligomers acting at the hypothalamus (through a mechanism involving NF-κB signaling) trigger peripheral IR, potentially establishing a second feed-forward loop between AD pathology, peripheral IR and brain IR (Clarke et al., 2015).

Aβ can be degraded by a variety of peptidases, such as the insulin degrading enzyme (IDE), neprilysin and angiotensin converting enzyme, as well as multiple serine proteases (plasmin, urokinase-type and tissue-type plasminogen activators; Wang et al., 2006; Saido and Leissring, 2012). Because of IDE’s ability to degrade insulin as well as Aβ42, it is thought to be a link connecting hyperinsulemia, IR, and AD (Authier et al., 1996; Qiu and Folstein, 2006). Although IDE is thought to only cleave monomeric Aβ (Hulse et al., 2009; Saido and Leissring, 2012), a decrease in its action could shift the equilibrium towards Aβ oligomerization. In mice, IR leads to increased brain amyloidosis through an increase in gamma-secretase activity, as well as decreased IDE (Ho et al., 2004; Starks et al., 2015). Furthermore, in AD patients with the APOE ɛ4 allele, IDE expression in areas such as the hippocampus is greatly reduced (Edland, 2004).

## The Interplay of Insulin Resistance and Tau Pathology

Tau is a member of a large group of proteins known as microtubule associated proteins (MAPs). In its native conformation, tau is a soluble and unfolded protein involved in microtubule stabilization and axonal outgrowth. However, hyperphosphorylated tau tends to aggregate and these tau aggregates are seen in various neurodegenerative diseases. In AD, tau forms intracellular NFTs, which alongside extracellular Aβ NPs constitute the two main histopathological hallmarks of the disease (Brandt and Leschik, 2004).

Several studies have implicated IR in tau aggregation, which largely depends on its phosphorylation state, which is in turn determined by the balance between various kinase and phosphatase activities. Intravenous insulin administration exerts a biphasic effect on tau phosphorylation. Short-term administration of insulin to human neuroblastoma cells or rat primary cortical neurons leads to rapid hyperphosphorylation of tau at several Ser/Thr residues, whereas prolonged exposure results in decreased phosphorylation (Lesort et al., 1999; Lesort and Johnson, 2000). This increase and subsequent decrease is mirrored by the activity of GSK-3β, widely considered to be the primary kinase responsible for the phosphorylation of Tau *in vivo* and modulated by insulin via the PKB/Akt pathway (Welsh and Proud, 1993; Hong and Lee, 1997; Planel et al., 2002; Llorens-Martín et al., 2014). A recent study suggests that a shift in APP processing from the α-secretase pathway to the β- and γ-secretase pro-amyloidogenic pathway increases GSK-3β-mediated tau phosphorylation, establishing a connection between the two core pathologies in AD (Deng et al., 2015), with brain IR aggravating them both. Upstream of GSK-3β, PKB/Akt itself also functions as a Ser/Thr kinase and has the ability to phosphorylate Tau directly, at least *in vitro* (Ksiezak-Reding et al., 2003; Zhou et al., 2009). Conversely, inhibiting the Ser/Thr phosphatases responsible for tau dephosphorylation can also increase the overall phosphorylation of tau. Protein phosphatase 2 (PP2A) is the primary tau phosphatase implicated in AD and is suppressed by insulin administration in both human and animal studies (Gong et al., 1995; Kins et al., 2001; Vogelsberg-Ragaglia et al., 2001; Clodfelder-Miller et al., 2006; Papon et al., 2013). Ob/ob transgenic mice are obese with high blood sugar and insulin levels, low levels of IRS-1 and 2, behavioral deficits, and tau hyperphosphorylation (Kerouz et al., 1997; Asakawa et al., 2003; Kim et al., 2013; Porter et al., 2013). Db/db mice also reliably display a phenotype of obesity, increased tau phosphorylation and IR accompanied by profound behavioral deficits in learning and memory (Kim et al., 2009; Sharma et al., 2010; Dinel et al., 2011). The combined effects of diminished insulin pathway activity in increasing tau phosphorylation and decreasing tau de-phosphorylation may broadly explain the increased tendency for tau aggregation with brain IR. Moreover, in the brains of AD patients, increased cytosolic levels of IRS-1 pS312 and pS616 correlate with the presence of NFTs, whereas, in CN controls, IRS-1 pS312 is restricted to nuclear regions of the cell. This finding suggests that IRS-1 phospho-species may have actions promoting tau pathology in AD beyond their role in the development of brain IR (Moloney et al., 2010).

Besides its role in the development of Aβ and tau pathology, brain IR can also directly affect synaptic function and cognition. For instance, in mice, down-regulation of insulin receptors in the hippocampus impairs hippocampal long-term potentiation and spatial learning (Grillo et al., 2015), whereas their down-regulation in the hypothalamus results in decreased hippocampal brain derived neurotrophic factor (BDNF; Grillo et al., 2011). Neurodegeneration, tau hyperphosphorylation and increased Aβ burden have also been reliably evoked in transgenic mice as a consequence of HFD, an intervention that reliably causes IR (Julien et al., 2010; Hiltunen et al., 2012). Both IR and oxidative stress independently lead to the accumulation of Aβ and phosphorylated tau (Chen et al., 2003; Grünblatt et al., 2007). Oxidative stress, an imbalanced biochemical state wherein the cell produces more reactive oxygen species than its antioxidant activity can withstand, also occurs as a result of metabolic syndrome and obesity (Davì et al., 2002).

## Spatial Correlation of IR-Related Genes and AD Pathology

The emergence of “big data” in neuroscience, particularly from gene expression microarrays, brought with it promising bioinformatics methods designed to take advantage of its sheer volume. The prominence of these new “neuroinformatics” methods in no way implies that older, well established data should be left behind. The synthesis of old and new data can be invaluable for exploratory studies and hypothesis generation. For example, when data of different modalities are distributed spatially over the entire brain, as is often the case for MRI data, tissue histology, positron emission tomography (PET), etc., this opens the possibility of comparison by pairwise spatial correlation. Often referred to as “guilt by association”, (Stuart et al., 2003), the concept behind this method is that shared spatial patterns of gene expression and other data (e.g., MRI features) also suggests participation in a shared function (Hawrylycz et al., 2011). This type of analysis essentially examines genotype-phenotype associations across small parcels of the brain rather than across human subjects. This approach may be suitable for diseases where there is a concrete spatial pattern of vulnerability across brain areas and for testing hypotheses that associate preferential vulnerability and differential gene expression.

**Figure 1:**
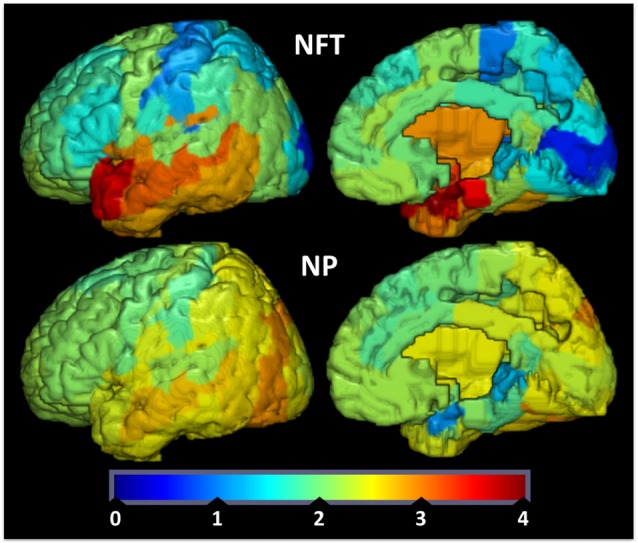
**Tau tangle (neurofibrillary tangles (NFT), upper row) and amyloid-beta plaque (neuritic plaques (NP), bottom row) values were redrawn from data originally presented in Arnold et al. (1991) and superimposed on Brodmann maps (BA 1–48) from MRIcroGL version 1.150909.** NFT and NP values are double-blinded rater assessments of tangle or plaque density. Color map and bar (“jet”) is red high, blue low.

**Figure 2:**
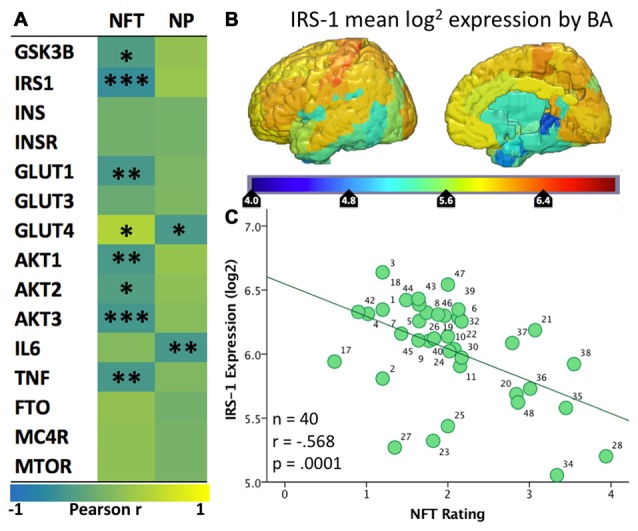
**(A)** Heatmap (“jet”: red high, blue low) of the spatial correlation between levels of expression of various genes from the Allen Human Brain Atlas and the density of tangles (NFT) or plaques (NP) from Arnold et al. (1991). Asterisks (*/**/***) represent *p* values of <0.05/.01/.001, respectively. **(B)** Map of mean IRS-1 log2 expression in the six healthy human specimens included in the Allen Human Brain Atlas. **(C)** Scatter plot of the mean IRS-1 log2 expression from the Allen Human Brain Atlas and the density of tangles (NFT) from Arnold et al. (1991). Each of the 40 data points corresponds to a BA for which both gene expression levels and tangle density ratings were available.

To provide further evidence for the relationship between brain IR and the propensity to develop plaques and tangles., we examined how the spatial distributions of Aβ-containing NPs and hyperphosphorylated tau-containing NFTs relate to the spatial expression of genes implicated in brain IR. We hypothesized that areas that show lower levels of GLUT and insulin signaling genes are less able to adapt to energetic challenges and are more vulnerable to AD pathology (Mamelak, 2012). We derived values of double-blinded rater assessments of the density of plaques and tangles from a seminal histological study on their topography in AD (Arnold et al., 1991) and converted them into 3D spatial map in Montreal Neuroimaging Institute (MNI) space (Figure 1). We then derived microarray expression levels (log^2^) for IR-related genes of interest (GSK3B, IRS1, INS, INSR, GLUT1, GLUT3, GLUT4, AKT1, AKT2, AKT3, IL6, TNF, FTO, MC4R and mTOR) from the Allen Human Brain Atlas (AHBA^1^), using the single probe with the highest overall expression when multiple probes exist. Like the NP/NFT maps, the AHBA provides numerous (~500 per specimen) microarray samples spatially distributed over six healthy “normal” control brain specimens (Hawrylycz et al., 2012; Sunkin et al., 2013). The expression levels for brain samples located within a given Brodmann area were averaged to make a new 3D map for each gene probe registered into MNI space and broken down by Brodmann areas. With these two spatially coregistered maps of histopathological and AHBA gene expression data established, a custom MATLAB (The Mathworks, Inc., Natick, MA, USA) script was used to perform pairwise Pearson correlations between NP or NFT densities vs. gene expression values for each Brodmann area (Figure 2).

Given that the AHBA brain specimens belonged to healthy individuals, positive correlations indicate regions where the normal expression of these genes is spatially similar to the Tau and/or Aβ pathologies seen in AD. In other words, these genes have higher expression in regions with high density of plaques or tangles in AD and lower expression in less vulnerable regions. A strong and significant positive association was seen between NFT density and expression of GLUT4 (*r* = 0.39, *p* = 0.018). Negative correlations suggest the reverse; wherein normal expression of these genes is low in the areas most vulnerable to AD plaques and tangles and high in less vulnerable areas. Significant negative correlations with NFTs were found for multiple insulin signaling genes, including IRS1 (−0.57, *p* < 0.001), AKT1 (*r* = −0.42, *p* = 0.007), AKT2 (*r* = −0.33, *p* = 0.033), AKT3 (*r* = −0.45, *p* = 0.003), GSK3B (*r* = −0.36, *p* = 0.019), and GLUT1 (*r* = −0.43, *p* = 0.005). The NP map correlated negatively with GLUT4 (*r* = −0.42, *p* = 0.01).

**Figure 3:**
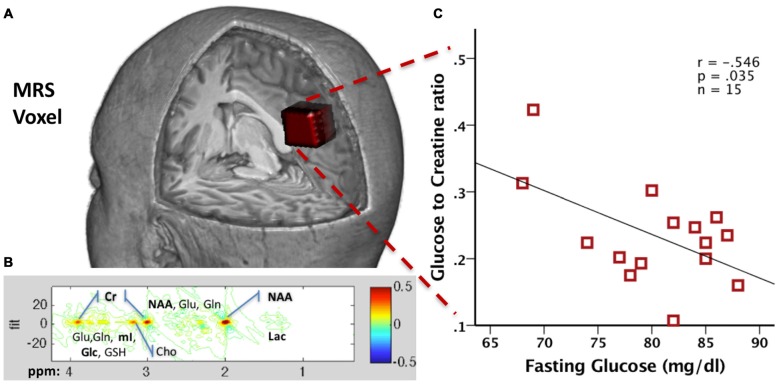
**(A)** Precuneal voxel placement for the junctional point-resolved spectroscopy (J-PRESS) acquisition is shown in red (25 × 18 × 20 mm^3^) within a 3D brain cutaway image (figure created in MRIcroGL version 1.150909). **(B)** Sample 2D J-PRESS spectral fitting from a representative 48-year-old male cognitively normal (CN) participant. **(C)** Scatter plot of the correlation between the Glc/Cr and fasting Glucose values in 15 healthy male participants (red squares).

IRS-1 regulates insulin signaling upstream of AKT and GSK3B, and prior studies have noted a decreased overall level of IRS-1 and related pathway molecule expression in AD neurons (Steen et al., 2005; Moloney et al., 2010). The observed negative spatial correlation with NFTs suggests that regions that normally show low levels of expression of IRS-1 are more likely to develop tau pathology in the setting of AD. We recently published a study showing that levels of pSer312-IRS1 in extracellular vesicles (EVs) enriched for neuronal origin are associated with brain atrophy in a regional pattern that corresponds to IRS1 expression (Mullins et al., 2017). Given that NFTs are known to be closely associated with atrophy, our findings collectively tie together IRS1 expression and post-translational phosphorylation, NFT pathology and atrophy. Regarding GLUTs, it has already been reported that neurons in areas vulnerable to AD show partial GLUT4 dependence, and it has been suggested that this may partially explain their vulnerability (Morgello et al., 1995; Apelt et al., 1999; Duelli and Kuschinsky, 2001). Moreover, we have noted that different isoforms of GLUT1 are expressed by astrocytes and endothelial cells, but unfortunately it is unclear to what extent GLUT1 expression in AHBA samples represents astrocytes vs. endothelial cells. Nevertheless, the present analysis demonstrates that normal regional expression of GLUT4 is positively associated with NFT density in AD, while GLUT1 is negatively associated. In other words, areas that normally have few GLUT1s and many GLUT4s show the greatest propensity for developing tau pathology in AD; see Figure 3 for summary and select detailed scatterplots from these findings. For the IR genes of interest, there are more (8 vs. 2) correlations with the NFTs than the NPs map.

## IR as A Link Between Aβ and Tau Pathologies in AD

One of the main enduring mysteries in AD is the different distribution of NFTs and NPs in the disease (Arnold et al., 1991). The various lines of evidence reviewed above and the novel analysis presented enable us to formulate a bold new hypothesis that considers IR as an important link between Aβ and Tau pathologies in AD and the main determinant of their regional distribution. Baseline differences in the reliance in glycolysis to generate energy, the expression of GLUT and insulin signaling genes determine the vulnerability of different brain regions to Tau and/or Aβ pathology. As mentioned already, extensive temporo-parietal areas of the brain show significant metabolic reliance on glycolysis (Phelps and Barrio, 2010; Vaishnavi et al., 2010; Vlassenko et al., 2010), which generates lactate. High lactate is associated with high interstitial Aβ, which assembles into Aβ oligomers. These Aβ oligomers promote Ser phosphorylation of IRS-1, impeding downstream insulin signaling and leading to brain IR. A feed-forward loop is established between IR and Aβ pathology leading to progressive Aβ deposition in NPs across extensive parts of the brain. Chronic IR promotes tau hyperphosphorylation and this effect is more pronounced in regions that show low expression of insulin signaling proteins (IRS-1, Akt, etc.) at baseline (earlier adult life). As a result, hyperphosphorylated tau leads to the development of NFTs in a different and more restricted regional pattern than Aβ. The sum of these three inter related pathologies (IR, Aβ, Tau) produces Alzheimer’s disease (AD).

This hypothetical paradigm is based on correlational studies but its predictions are testable (and falsifiable). For instance, it can be falsified by examining the spatial correlation of IR-related gene expression and Aβ and Tau pathologies in brain specimens from subjects across the spectrum from normal cognition to AD. Moreover, it allows for novel predictions that can be tested in clinical trials. For instance, interventions that increase insulin sensitivity (see below) may be expected to decrease the rate of Aβ production and tau hyperphosphorylation. For these predictions to be testable though, biomarkers that reflect AD pathogenic processes, brain metabolism and IR are required.

## Traditional and Novel Biochemical Measures of Insulin Resistance

The traditional gold standard for measuring systemic IR is the hyperinsulinemic euglycemic clamp, as this technique provides highly reproducible data with a distinct physiological meaning (DeFronzo et al., 1979). Unfortunately, the technique is procedurally complex and requires considerable expertise to obtain reliable results (Le et al., 2009). HOMA-IR, as well as its most recent version HOMA2-IR, provide an estimate of systemic IR and β cell function by combining fasting insulin and glucose levels in a single metric (Matthews et al., 1985). IR as measured by HOMA-IR (see below) has been shown to correlate with increased CSF levels of AD biomarkers such as soluble amyloid precursor protein β (sAPPβ), p-tau181 and Aβ42 (Starks et al., 2015; Hoscheidt et al., 2016). Unfortunately, HOMA-IR is subject to measurement errors especially if a single blood sample is used and is also susceptible to physiological fluctuations in fasting glucose and insulin levels, limiting its reliability. Moreover, HOMA-IR does not distinguish between brain-specific and systemic IR, making the search for biomarkers directly reflecting brain phenomena imperative for studying the role of IR in AD.

EVs are membranous particles and are secreted from nearly every cell type throughout the body, whereas the term exosomes refers to a subtype of EVs from 30 nm to 150 nm in size that have been implicated in a variety of functions. EVs extracted from murine brain tissue have been shown to contain APP, as well as Aβ species (Bellingham et al., 2012; Perez-Gonzalez et al., 2012) Moreover, secreted exosomes have been shown to contain hyperphosphorylated tau as well as Aβ (Rajendran et al., 2006). Interestingly, EVs also contain proteolytically active IDE which may degrade extracellular Aβ (Bulloj et al., 2010).

Whereas these and subsequent findings implicated EVs in AD pathogenesis, we are primarily interested in EVs as a source of biomarkers for the disease. Our team has been a pioneer in isolating plasma EVs enriched for neuronal origin. To date, AD biomarkers derived from neuronal origin-enriched EVs include not only the main pathogenic proteins (p-tau and Aβ42) but also intracellular signaling molecules, such as phosphorylated IRS-1, Cathepsin-D, REST, LRP6, and others (Fiandaca et al., 2015; Goetzl et al., 2015a,b; Kapogiannis et al., 2015). Of particular interest for the study of brain IR are our findings concerning IRS-1. In plasma EVs enriched for neuronal origin, we measured total, pSer312- and p-PanY- (pan-Tyr phosphorylated) IRS-1 in a clinical cohort of AD patients and CN older control subjects (as well as patients with Frontotemporal Dementia, as a neurodegenerative disease control, and CN patients with diabetes, as a metabolic disease control). We showed that these two phospho-species, as well as their ratio, were highly significantly different in AD patients vs. all control groups. Interestingly, subjects with diabetes had intermediate values between AD patients and CN controls, suggesting that the peripheral IR that characterizes diabetes is linked to some degree to brain IR and corroborating the extensive body of literature suggesting that IR and diabetes are risk factors for AD, but by no means obligatory causative factors. Furthermore, IRS-1 phospho-species achieved remarkable classification accuracy for AD patients vs. controls, and in a separate smaller cohort were already abnormal up to 10 years before clinical onset of AD (Kapogiannis et al., 2015).

In a recent study Mullins et al. (2017), we showed that, in a cohort of AD patients without systemic IR, pSer312-IRS-1 was positively associated with MRI atrophy, whereas p-PanY-IRS-1 was negatively associated with it, in a highly characteristic pattern of regions. The significance of this regional pattern lies in its spatial correlation with the normal IRS-1 brain expression. We speculate that neuronal-enriched plasma EVs containing IRS-1 may be preferentially derived from brain regions with high levels of IRS-1 expression. Therefore, the IRS-1 phosphorylation pattern seen in these EVs may reflect its phosphorylation status in specific brain regions that suffer brain atrophy in early AD in association with higher burden of brain IR. Interestingly, systemic IR (either in terms of fasting insulin or HOMA-IR) showed no associations with regional AD atrophy, further suggesting that EV-based biomarkers are well- suited as a tool for investigating brain IR in AD. These findings not only further establish the links between IR and AD, but provide hope for a blood-based diagnostic assay to identify individuals who would likely develop AD preclinically. Importantly, since interventions that aim to reverse brain IR in AD are being subjected to clinical trials (e.g., intranasal insulin, exenatide), using these biomarkers we may be able to demonstrate target engagement and follow response to treatment.

## Neuroimaging Studies of Insulin Resistance

Fluorodeoxyglucose Positron emission tomography (FDG-PET) imaging has long been considered the definitive method for assessing brain metabolism. FDG is an analog of glucose that is imported in cells in a similar fashion to glucose that provides a reliable estimate of the cerebral metabolic rate for glucose (CMRGlc). As was initially shown in 1989 (Friedland et al., 1989) and replicated in numerous cohorts since (Herholz et al., 2002; Langbaum et al., 2009), CMRGlc is decreased in AD with a characteristic regional pattern over the medial/lateral parietotemporal and frontal cortices. Intriguingly, the same pattern of relative hypometabolism was shown in relation to HOMA-IR in CN post-menopausal women (Rasgon et al., 2014), older adults with prediabetes/T2D (Baker et al., 2011), and those at higher risk for AD given their parental history (Willette et al., 2015a). This suggests a continuum of vulnerability of glucose metabolism in these conditions that culminates in clinical AD. In a study of patients with MCI and AD, we showed that HOMA-IR is negatively associated with glucose metabolism in brain areas vulnerable to AD pathology, but not in areas typically unaffected by AD (Willette et al., 2015c). In addition, we showed that HOMA-IR is paradoxically (and perhaps maladaptively) positively associated with hippocampal glucose metabolism in MCI patients prior to conversion to AD dementia (Willette et al., 2015c).

Conflicting findings exist on the relationship between Aβ deposition and peripheral IR in PET studies using Pittsburgh compound B (PiB) or Florbetapir (F18-AV-45), with some studies showing no such relationship (Edison et al., 2007; Thambisetty et al., 2013) and others indicating a relationship for normoglycemic but not hyperglycemic CN older adults (Willette et al., 2015b). The recent development of tau-PET imaging has attracted a surge of interest due to recent findings that it presents a stronger relation to neurodegeneration and cognitive decline than Aβ (Sarazin et al., 2016; Thal and Vandenberghe, 2016), but being a very recent development there are no published results to report on the relation of tau distribution to IR.

Structural and functional MRI have also been used to study IR-AD associations. In late middle-aged, cognitively healthy individuals, HOMA-IR has been negatively associated with hippocampal (Rasgon et al., 2011) and cortical gray matter (Willette et al., 2013) volumes in a pattern characteristic of AD. Diffusion MRI has revealed deficits in the microstructural integrity of gray and white matter in AD (Meng et al., 2012; Hong et al., 2013; Molinuevo et al., 2014; Weston et al., 2015) and type 2 diabetes (Hsu et al., 2012; Reijmer et al., 2013; Xiong et al., 2016) that are associated with impaired cognitive performance. Functional MRI (fMRI) has been used to demonstrate that insulin infusion enhances activity in the medial temporal lobe (Zhao and Townsend, 2009), that middle-aged CN subjects with peripheral IR (Kenna et al., 2013) or type 2 diabetes (Musen et al., 2012; Chen et al., 2014) show impaired functional connectivity of the hippocampus and the default mode network, Advanced two-dimensional (2D MRS) methods currently gaining favor are capable of detecting glucose concentrations within specific regions of the brain (Thomas et al., 2003) and may be used some day to study IR in relation to AD. An example of this method is provided below.

As a general comment to all neuroimaging studies to date; since no good biomarker of brain IR existed, the field had to rely on the assumption that some peripheral IR measure (such as HOMA-IR) can be used as a surrogate of brain IR. With the discovery of IRS-1 phospho-peptides in neural-origin plasma EVs (Kapogiannis et al., 2015) and the demonstration of their neuroimaging correlates (Mullins et al., 2017), we have introduced biomarkers for brain-specific IR. We hope that future neuroimaging studies will take advantage of these novel biomarkers and examine more brain-specific associations.

## Future Directions: Glucose Measurement Via 2D Magnetic Resonance Spectroscopy

Modern magnetic resonance spectroscopy (MRS) techniques have recently advanced to the point of reliable measurement of *in vivo* glucose levels in the brain. Earlier 1D MRS methods had difficulty quantifying the glucose metabolite signal due to the presence of multiple overlapping signals in the acquired spectra (Steinberg and Velan, 2013). The method of 2D junctional point-resolved spectroscopy (J-PRESS) solves this problem by sampling the signal at multiple echo times, which adds another dimension to the spectra to increase specificity and reduce overlapping peak tails (see Figure 3B for spectral fitting; Schulte and Boesiger, 2006). This increased specificity is of particular interest for neurotransmitters and metabolites linked to AD pathogenesis, particularly glucose (Kapogiannis et al., 2013). The J-PRESS technique thus presents researchers with the opportunity to safely estimate the actual *in vivo* level of combined intracellular and extracellular brain glucose. Therefore, this measure may complement information acquired with FDG-PET, which assesses the metabolic rate of glucose rather than its concentration. In comparison to PET, MRS has the advantage of not using radiation and potentially being more widely available, since it requires only MR scanning rather than nuclear medicine capabilities.

To demonstrate technical feasibility and provide proof of concept for this method, we collected JPRESS data from a set of 15 fasting healthy male volunteer participants (40.5 ± 7.8 years old) who also underwent a blood draw for fasting glucose. Both the MRS and the blood draws took place after an 8-h fast. Plasma samples were processed via YSI 2300 STAT PLUS™ Glucose analyzer (YSI Inc., Yellow Springs, OH, USA) to derive glucose concentration. A 2D J-PRESS acquisition with maximum-echo sampling was used to acquire metabolite concentrations from a bilateral anisotropic precuneus Voxel (25 × 18 × 20 mm^3^, see Figure 3A), also used in our previous study (Kapogiannis et al., 2013). ProFit software (Schulte and Boesiger, 2006) was used to acquire the linear combinations of simulated basis metabolite spectra to generate relative concentrations to creatine. All data were acquired as part of the visit for a physiology study on glucose metabolism (ClinicalTrials.gov Identifier NCT01517100) and approved by the Institutional Review Board of the National Institute of Diabetes and Digestive and Kidney Diseases, Bethesda, MD, USA. All participants provided written informed consent.

We found that glucose concentration was reliably measured in all 15 subjects, with a mean Cramer-Rao lower bound (crlb%, a measure of signal reliability) of 13.7%, ranging from 7.6% to 21.8%. In addition, we found a moderate negative correlation between MRS Glc brain concentrations and fasting glucose levels (*r* = −0.546, *p* = 0.035; Figure 3C). The exact significance of this finding and its pertinence to disease states is the subject of ongoing research. As the MRS acquisition spans about 24 min, MRS Glc likely reflects steady state glucose levels. One possible explanation is that higher brain MRS Glc results from lower brain glucose metabolism. It is known that, during prolonged fasting (after 12 h), the body (and the brain) switches from glucose to ketone metabolism (Foster, 1967). It has been shown that ketones are the preferred energy source by the brain, since the higher their plasma concentration, the higher their uptake by the brain, and the higher their percent contribution to total brain energy metabolism (Cunnane et al., 2016a,b). Subjects with lower fasting plasma glucose during prolonged fasting may have switched more fully into brain ketone metabolism, with a corresponding decrease in brain glucose metabolism and increase in MRS Glc concentration. This hypothesis will be tested in an ongoing clinical study on the brain effects of intermittently prolonged calorie restriction (5–2 calorie restriction), which examines the effects of the diet on MRS glucose vis a vis levels of metabolites in CSF and plasma (NCT02460783). More broadly, this technique opens yet another window into brain metabolism for examining the effects of disease states, such as AD.

## Clinical Trials Targeting Brain IR in AD and Biomarkers

There are several approaches for targeting brain IR as a therapeutic strategy for AD. Perhaps, the most straightforward one is to try to overcome brain IR by increasing brain availability of insulin. Since systemic insulin administration in non-diabetic subjects produces hypoglycemia, the approach that has been promoted to achieve this goal is intranasal insulin administration, which involves bulk flow through the olfactory bulb into the brain (Born et al., 2002). In a Phase II clinical trial, patients receiving intranasal insulin for 4 months showed better cognition (especially memory) compared to those receiving placebo (Craft et al., 2012). A recent study using the long-acting insulin analog detemir via intranasal administration also yielded promising results (Claxton et al., 2015). A different strategy is brain insulin sensitization; two insulin-sensitizing drugs, rosiglitazone and pioglitazone, are currently being investigated as therapeutic agents for AD. Rosiglitazone potentiates the protective effects of insulin on cultured neurons and inhibits the production of Aβ42 in mice, but human trials have yielded disappointing results (Landreth et al., 2008; De Felice et al., 2009; Miller et al., 2011). In mice, pioglitazone improves learning, reduces tau and Aβ deposits in the hippocampus, and improves neuronal plasticity (Searcy et al., 2012). In humans, consistent pioglitazone administration has been associated with decreased incidence of dementia, but clinical trials are lacking (Heneka et al., 2015).

Glucagon like peptide 1 (GLP1) agonists have been shown to offer neuroprotection (Perry et al., 2002, 2007), reverse brain IR (Bomfim et al., 2012; Talbot and Wang, 2014), decrease Aβ and tau levels and deposits (Li et al., 2010; McClean et al., 2011), and decrease tau hyper-phosphorylation (Xu et al., 2015) in multiple cellular and animal models of AD. The GLP-1 agonist exenatide, has been shown to alleviate brain IR in AD by modifying the pattern of IRS-1 phosphorylation (Bomfim et al., 2012) and be neuroprotective against a variety of neurodegenerative diseases and stroke (Li et al., 2009; Martin et al., 2009; Tweedie et al., 2012). Importantly, exenatide has already demonstrated clinical effectiveness for Parkinson disease in terms of motor and cognitive performance measures (Aviles-Olmos et al., 2013). Our team recently completed a pilot clinical trial of exenatide in MCI/early AD (NCT01255163). Recently, it was reported that AD patients treated with another GLP-1 agonist, liraglutide, for 6 months showed a non-significant trend for increased CMRglc compared to placebo-treated patients. Importantly, the rate of progressive Aβ deposition in PiB PET was not affected by the treatment. The authors note that the findings are inconclusive in regard to the therapeutic potential of liraglutide, and by extension of GLP1 agonists, in AD (Gejl et al., 2016). In our view, this inconclusiveness stems from the limited relevance of the outcome measures to the mechanism of action of the intervention. Relying on cognitive/clinical outcomes or even biomarker outcomes far down-stream in the pathogenic cascade and relevant only to one particular aspect of disease pathogenesis (such as PiB PET) irrespective of the particular mechanism involved has plagued clinical trials in AD and prevented the field from extracting generalizable conclusions from the failures in individual clinical trials.

We are currently engaged in the analysis of IRS-1 phosphotypes and downstream signaling molecules in neuronal EVs from plasma samples from the pilot trial of exenatide in AD conducted at the National Institute on Aging (NIA) and several other clinical trials targeting IR. We are hopeful that a response of EV-based biomarkers and/or MRS Glc to experimental interventions would demonstrate mechanism-specific target engagement. In addition, if these interventions decrease Aβ production and tau phosphorylation, they would provide significant mechanistic support to the hypothetical paradigm advocated in this article.

## Conclusions

**Figure 4:**
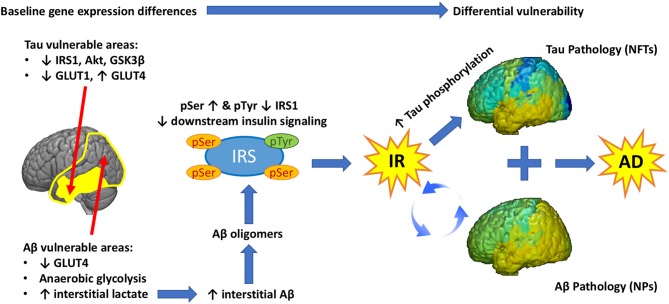
**Graphical abstract.** Baseline differences in the expression of glucose transporters (GLUT) and insulin signaling genes determine the vulnerability of different brain regions to Tau and/or Aβ pathology. Extensive temporo-parietal areas of the brain show significant metabolic reliance on glycolysis, which generates lactate. High lactate is associated with high interstitial Aβ, which assembles into Aβ oligomers. These Aβ oligomers promote Ser phosphorylation of IRS-1, impeding downstream insulin signaling and leading to brain IR. A feed-forward loop is established between IR and Aβ pathology leading to progressive Aβ deposition in NP. Chronic IR promotes tau hyperphosphorylation and this effect is more pronounced in regions that show low expression of insulin signaling proteins (IRS-1, Akt, etc.) at baseline. As a result, hyperphosphorylated tau leads to the development of NFT in a different and more restricted regional pattern than Aβ. The sum of these three inter related pathologies (IR, Aβ, Tau) produces Alzheimer’s disease.

This article attempted to disentangle the complex mechanisms underlying brain IR, highlight proven or plausible links to Aβ and tau pathologies in AD, as well as provide information about promising recent EV-based biomarkers, *in vivo* glucose MRS measures, and gene array neuroinformatics techniques. The convergence of such diverse sources of evidence makes it all but certain that brain IR plays a major role in AD pathogenesis linking the two main types of pathology (Figure 4). Ultimately, the merit of this hypothesis rests on demonstrating effectiveness in ongoing and future clinical trials.

## Author Contributions

RJM, TCD, CWC and DK wrote and edited the manuscript; RJM, TCD and DK were responsible for experimental design and conception; and RJM performed the analyses.

## Conflict of Interest Statement

The authors declare that the research was conducted in the absence of any commercial or financial relationships that could be construed as a potential conflict of interest.

## Footnotes

^1^http://human.brain-map.org/static/download

## Abbreviations

Aβ: Amyloid beta
AHBA: Allen human brain atlas
AD: Alzheimer’s disease
APOE: apoliproprotein-E
APP: amyloid precursor protein
AKT: Protein Kinase B
BA: Brodmann’s area
BMI: body-mass index
BBB: blood brain barrier
BDNF: brain derived neurotrophic factor
CAA: cerebral amyloid angiopathy
CSF: cerebrospinal fluid
cGMP: cyclic guanosine 3′,5′-monophosphate
CMRGlc: cerebral metabolic rate for glucose
CRLB: Cramer-Rao Lower Bound
CSVD: cerebral small vessel disease
EV: extracellular vesicles
FDG-PET: Fluorodeoxyglucose Positron emission tomography
FFA: free fatty acids
FTO: fat mass and obesity-associated protein
GLP: glucagon like peptide
GLUT: glucose transporter
GSK: glycogen synthase kinase
HFD: high fat diet
HOMA-IR: homeostatic model assessment for insulin resistance
IDE: insulin degrading enzyme
IL6: interleukin 6
INS: insulin
INSR: insulin receptor
IR: insulin resistance
IRS: insulin receptor substrate
ISF: interstitial fluid
JNK: c-Jun N-terminal kinase
J-PRESS: 2D junctional point-resolved spectroscopy
MAP: microtubule associated proteins
MCI: mild cognitive impairment
MC4R: melanocortin-4 receptor
MNI: Montreal Neuroimaging Institute
mTOR: mammalian target of rapamycin
MRI: magnetic resonance imaging
MRS: magnetic resonance spectroscopy
NF-kB: nuclear factor kappa-light-chain-enhancer of activated B cells
NFT: neurofibrillary tangles
NO: nitric oxide
NP: neuritic plaques
PET: positron emission tomography
PI3K: phosphoinositide 3-kinase
PiB: Pittsburgh compound B
PP2A: protein phosphatase 2
PKB/Akt: protein kinase B
RAGE: receptor for advanced glycation end products
S6K1: ribosomal protein S6 kinase beta-1
TNF: tumor necrosis factor
VCID: vascular contribution of cognitive impairment and dementia.
